# MiR224-5p Inhibitor Restrains Neuronal Apoptosis by Targeting NR4A1 in the Oxygen-Glucose Deprivation (OGD) Model

**DOI:** 10.3389/fnins.2020.00613

**Published:** 2020-06-25

**Authors:** Ling-Ling Liu, Shan Qiao, Mei-Ling Wang, Huai-Kuan Wu, Yong-Xin Su, Ke-Mo Wang, Xue-Wu Liu

**Affiliations:** ^1^Department of Neurology, Qilu Hospital, Cheeloo College of Medicine, Shandong University, Jinan, China; ^2^Department of Neurology, Liaocheng People’s Hospital, Liaocheng, China; ^3^Department of Neurology, Shandong Provincial Qianfoshan Hospital, The First Hospital Affiliated With Shandong First Medical University, Jinan, China; ^4^Department of Neurology, Binzhou Medical University Hospital, Binzhou, China

**Keywords:** middle cerebral artery occlusion, oxygen-glucose deprivation, miR-224-5p, nuclear receptor subfamily 4 group A member 1, apoptosis

## Abstract

This study was designed to investigate the molecular mechanism of stroke and to explore the effect of miR-224-5p in hypoxic cortical neurons. Firstly, we established a middle cerebral artery occlusion (MCAO) model with Sprague–Dawley rats. Triphenyltetrazolium chloride (TTC) staining showed the brain infarction of an MCAO rat. Longa scores of rats were significantly increased in 12th, 24th, and 48th hours after MCAO. Then, we found that miR-224-5p was increased after MCAO in rats by qRT-PCR. In order to investigate the effect of miR-224-5p in hypoxic neurons, we established an oxygen-glucose deprivation (OGD) model with cortical neurons. MiR-224-5p was also upregulated in neurons after OGD by qRT-PCR. After transfection of the miR-224-5p inhibitor, the number of neurons in the anti-miR-224-5p group significantly increased (*P* < 0.01) in comparison to the anti-NC group. Furthermore, Tuj1^+^ (neuronal marker) staining and TUNEL assay (to detect apoptotic cells) were performed in neurons. The survival of neurons in the anti-miR-224-5p group was significantly improved (*P* < 0.01), while the apoptosis of neurons in the anti-miR-224-5p group was significantly decreased (*P* < 0.01), when compared with that of the anti-NC group. In addition, we predicted that potential target genes of miR-224-5p were nuclear receptor subfamily 4 group A member 1 (NR4A1), interleukin 1 receptor antagonist (IL1RN), and ring finger protein 38 (RNF38) with bioinformatics databases, such as TargetScan, miRDB, miRmap, and miRanda. The result of qRT-PCR confirmed that NR4A1 was significantly decreased after hypoxic injury (*P* < 0.01). Meanwhile, luciferase reporter’s assay indicated that NR4A1 was the direct target of miR-224-5p. Compared with the anti-miR-224-5p + siNC group, the number of cortical neurons and the length of the neuron axon in the anti-miR-224-5p + si-NR4A1 group were significantly decreased (*P* < 0.01), and the number of neuronal apoptosis in the anti-miR-224-5p + si-NR4A1 group was increased (*P* < 0.01). In conclusion, miR-224-5p played a crucial role in hypoxic neuron injury through NR4A1, which might be an important regulatory mechanism in OGD injury of neurons.

## Introduction

Stroke is caused by cerebral ischemia and hypoxia, which can severely affect the health and has high mortality and morbidity (Michalski et al., 2011; Ferrell et al., 2014; Sun et al., 2017). Meanwhile, cerebral ischemia can also cause many remote organ dysfunctions (Klempt et al., 1992; Perlman, 2006). Due to its rising overall incidence in recent years, stroke has become an important disease of research. However, the specific mechanism of stroke is not entirely clear and no specific treatment is available. Therefore, the causal mechanism of stroke requires further investigation.

MicroRNAs (miRNAs) are a new class of non-coding RNAs, approximately 20 ∼ 28 nucleotides in length, which are encoded as short inverted repeats in the genomes of invertebrates and vertebrates (Moss, 2002; Shi, 2003; Son et al., 2014). As important regulators of gene expression at the post-transcriptional level (Bartel, 2004), miRNAs have complex and diverse regulatory functions in cells (Cui et al., 2006). They are involved in a variety of biological processes, such as proliferation, differentiation, metabolism, and cell apoptosis (Ambros, 2004; Bagga et al., 2005; Nohata et al., 2011). miR-224-5p is one of the members of the small non-coding RNA, which resides in the third intron of the GABAA receptor subunit (GABRE) gene on ChrXq28 (Wilke et al., 1997; Wang and Lee, 2011). The previous study demonstrated that miR-224-5p was likely to be co-transcribed with its host gene GABRE, which could be repressed by cooperation of p53 (White et al., 2010; Liang et al., 2013). MiR-224-5p participates in multiple biological processes, including tumor cell proliferation, migration, invasion, and apoptosis (Zhang et al., 2013). The mechanisms can be associated with the transcription factor SMAD4, miR-224-5p/HOXD10/p-PAK4/MMP-9 signaling pathway, AKT activation, NF-κB inflammatory pathway, and so on (Yao et al., 2010; Scisciani et al., 2012; Li et al., 2014; Yu et al., 2014). Researchers also find miR225 as one of the important regulators in brain function. However, the molecular mechanism of miR-224-5p on hypoxic neuron is unclear.

In the present study, we investigate the effect of miR-224-5p on hypoxic cortical neurons and explore the related downstream molecular mechanism. Thus, inhibition of miR-224-5p, or/and activation of NR4A1 may be an ideal regimen for preventing and treating hypoxic neuron damage.

## Materials and Methods

### MCAO Model

Sprague–Dawley (SD) rats were provided by the Experimental Animal Center of Shandong University. The animal study agreement was legally approved by the Ethics Committee for Animal Experimentation of Shandong University and in accordance with the guidelines of the National Institutes for Animal Research. SD rats (male, 210 *g*) were randomly divided into sham group and middle cerebral artery occlusion (MCAO) group. MCAO rats were performed as previously reported (Bian et al., 2015). Briefly, the rat was anesthetized with chloral hydrate (3.6%, 1 mL/100 *g*, and intraperitoneal), and the right common carotid and right external carotid arteries of rats were isolated and coagulated. A nylon suture (0.24 mm, Cinontech, Beijing, China) with a round tip was inserted into the internal carotid artery through the external carotid artery stump and advanced to occlude the origin of the middle cerebral artery. The suture was removed at 2 h after occlusion. Sham-operated rats underwent the same surgical procedures without inserting suture.

### Neurological Deficit Assessment

The Longa score test was performed to determine whether the MCAO model was established successfully (Goldberg et al., 1997). In this study, Longa scores were used for evaluating the neurological function of rats in 12th, 24th, and 48th hours after MCAO. If the rat had no neurological defect, the score was 0. If the rat could not fully stretch its left forepaw, the score was 1. If the rat circled while walking, the score was 2. If the rat tumbled while walking because of hemiplegia, the score was 3. If the rat was unconscious and unable to walk, the score was 4.

### Real-Time Fluorescent Quantitative PCR (qRT-PCR)

Total RNA was extracted using TRIzol reagent (Gibco, Rockville, MD, United States). After DNAse I treatment, RNA was reverse transcribed with reverse transcriptase. The levels of miR-224-5p analyzed by qRT-PCR were normalized to that of U6. After that, RT-PCR was performed with the PCR Master Mix Kit (Fermentas, Burlington, VT, United States). MiR-224-5p and U6 were amplified through mixtures which included 12.5 μL 2 × PCR Master Mix (Takara), 0.5 μL upstream primer (Sangon, Shanghai, China), 0.5 μL downstream primer (Sangon, Shanghai, China), 10.5 μL PCR Nuclease-Free Water, and 1 μL cDNA. U6 and β-actin were used as endogenous controls, respectively. The primers used in the protocol were as follows: miR-224-5p, 5-ACAAGTCACTAGTGGTTCC-3 (forward) and 5-CAGTGATGTTGCGGTCTG-3 (reverse); U6, 5-CTCGCTTCGGCAGCACA-3 (forward) and 5-TGGTGTCG TGGAGTCG-3 (reverse); β-actin, 5-TCTACAATGAGCTGCG TGTG-3 (forward) and 5-GGTGAGGATCTTCATGAGGT-3 (reverse); nuclear receptor subfamily 4 group A member 1 (NR4A1), 5-TGATGTTCCCGCCTTTGC-3 (forward) and 5-CAATGCGATTCTGCAGCTCTT-3 (reverse); interleukin 1 receptor antagonist (IL1RN), 5-CTCAGCAACACTCCTAT-3 (forward) and 5-TCCTGGTCTGCAGGTAA-3 (reverse); ring finger protein 38 (RNF38), 5-CTCGAGGAATTCGCCACCATG GCTTGTAAGATATCTCCCGGG-3 (forward) and 5-GGATCC GCGGCCGCTCATTCTGAATCCCGATGCACTTC-3 (reverse). All reactions were performed for 40 cycles, and then the fluorescence value was recorded from every reaction. A real-time fluorescent quantitative machine, designated as ABI7300, was used in this process. Data were analyzed using a relative critical threshold (Ct) method where the amount of target was normalized to the amount of the internal control. Image analysis was done by Quantity One software (Bio-Rad Laboratories, Inc., Richmond, CA, United States).

### Culture of Primary Cortex Neurons

Primary cortical neurons were separated from neonatal SD rats within 1 day of birth. The obtained small cortical tissue was placed in a culture ware containing 2 mL DMEM (high-glucose) liquid. Cortices were harvested and cut into approximately 1-mm^3^ small pieces, then digested with 0.05% trypsin (Gibco, Rockville, MD, United States) at 37°C for 10 min. Then, the high-glucose DMEM complete medium containing 10% fetal bovine serum (FBS) was added to the culture ware to stop digestion. The tissue suspension was centrifuged at 1000 rpm for 10 min, resuspended, and plated in 96-well or 6-well plates (Corning, Corning, NY, United States) coated with poly-d-lysine and laminin (Sigma-Aldrich, St. Louis, MO, United States) at a density of 5 × 10^5^ cells/ml with a neurobasal medium. After this, half of the neurobasal medium was refreshed every 3 days.

### Transfection of MiR-224-5p Mimic/Anti-miR-224-5p in Neurons

We transfected the miR-224-5p mimic or anti-miR-224-5p in neurons in the fifth day of primary cortex neurons. Then, we established the OGD model in the eighth day of primary cortex neurons. We detected indexes in the ninth day of primary cortex neurons. Primary cortex neurons were cultured for 3 days with cells at 50–80% confluence prior to transfection. For the transplantation, primary cultured cortex neurons were divided into six groups: control (neurobasal medium only), OGD, OGD + mimic-NC (nonsense miRNA), OGD + anti-NC (nonsense miRNA), OGD + miR-224-5p, and OGD + anti-miR-224-5p group. The mimic and inhibitor of miR-224-5p were designed and synthesized by RiboBio (Guangzhou, China). Firstly, we mixed the buffer and miRNA and 3 μl of SuperFectin^TM^ II reagent was added to the mixture. Then, a mixture of miR-224-5p (50 nM) or anti-miR-224-5p (100 nM) was added drop-wise to the appropriate wells, respectively. The neurons were stored at 37°C for 24 h. Red Cy3-5′-fluorescence was observed with a fluorescence microscope (Leica, DM4000B, Wetzlar, Hesse-Darmstadt, Germany) 72 h.

### Construction of the OGD Model

In this study, the oxygen–glucose deprivation (OGD) model was established after 3 days of transfection miR-224-5p mimic/anti-miR-224-5p, which was performed as previously described (Milner et al., 2008). In short, the neurobasal medium was replaced with the medium that had no glucose, and neurons were placed into an incubator (Don Whitley Scientific, Waltham, Shipley, United Kingdom) with 0.1% oxygen, 95% N_2_, 5% CO_2_, at 37°C for 2 h. After 2 h, the culture medium was replaced by a neurobasal medium, and then the cells were put in the incubator at 37°C with 5% CO_2_ for 24 h. Then, we detected indexes, such as MMT, TUNEL, and Tuj1.

### MTT Assay

MTT assay was performed following the protocols (Xu et al., 2018). In brief, cells (5 × 10^5^ cells/ml) were seeded in 96-well plates that had been pre-coated with poly-L-lysine and cultured overnight in a CO_2_ incubator (95% air/5% CO_2_). The treated cells were visualized using a fluorescence microscope (Leica, DM4000B, Wetzlar, Hesse-Darmstadt, Germany) equipped with a digital camera. Then, cells were incubated in a serum-free medium containing MTT solution in the darkness for 4 h at 37°C. The MTT solution was discarded, and 100 μl DMSO was added to every well to dissolve the formazan crystals. The value of optical density was measured at a wavelength of 562 nm using a Bio-Rad microplate reader (Bio-Rad Laboratories, Inc., Richmond, CA, United States). Cell survival for each treatment was calculated as a percentage of the control.

### TUNEL Assay

The TUNEL reaction mixture of enzyme solution and labeling solution (*In situ* Cell Death Detection Kit, TMR red; Cat. NO. 12156792910) was added at a ratio of 1:9 (v/v). After adding the TUNEL reaction mixture, neurons were stored at 4°C overnight in the dark. Then, neurons were washed with PBS and were stained with DAPI for 5 min. Results were obtained under fluorescence microscopy (Leica, DM4000B, Wetzlar, Hesse-Darmstadt, Germany). Apoptosis was quantified by determining the percentage of TUNEL/DAPI using ImageJ software (National Institutes of Health, Bethesda, MD, United States).

### Immunofluorescence Staining of Tuj1 *in vitro*

Immunocytochemical analysis of Tuj1 was performed to detect the effect of miR-224-5p transfection on neurons. Briefly, for neuronal immunocytochemistry, neurons were directly permeated in PBS containing 3% goat serum for 30 min at 37°C. Then, the neurons were incubated with the primary anti-Tuj1 antibody (1:100, mouse, Abcam, Cambridge, Cambs, United Kingdom) and species-specific secondary antibody (1:100, mouse, Abcam, Cambridge, Cambs, United Kingdom) as described previously. The number of Tuj1^+^ cells quantified was normalized to the total DAPI to confirm the purity of the neurons. Then, numbers of Tuj1^+^ cells were counted per mm^2^ to compare the population changes of neurons in different groups by ImageJ software (National Institutes of Health, Bethesda, MD, United States). All detections were evaluated by 3 investigators blinded to the experimental information.

### Measurement of Axon Length in Neurons

Four days after the transfection of anti-miR-224-5p, images of these neurons were obtained under microscopy (Leica, DM4000B, Wetzlar, Hesse-Darmstadt, Germany). For measurements of the axon length of living neurons, images were randomly selected. Axon lengths were measured and were quantified. The mean axonal length was calculated from counts on a 6-well plate in each group with 5 images in each well, which were obtained from the upper, middle, lower, left, and right of each well, respectively. Three examiners who can distinguish the axon and dendrite easily were employed to measure the axon length, and all the examiners were blinded to the group identity.

### Luciferase Reporter Assay

The 3′-UTR of the NR4A1 mRNA-containing miR-224-5p-binding site was PCR-amplified and inserted downstream of a luciferase reporter gene in the pmirGLO vector. In addition, a mutant construct containing mutations within the binding sites was generated using the QuikChange^®^ site-directed mutagenesis kit (Agilent, Palo Alto, CA, United States) according to the manufacturer’s instructions. The cells were co-transfected with miR-224-5p mimics and wild-type or mutant luciferase reporter constructs or transfected with wild-type or mutant luciferase reporter Dual-Luciferase Reporter Assay System (Promega, Madison, WI, United States) according to the manufacturer’s instructions. Renilla luciferase intensity was normalized to firefly luciferase intensity. As for the binding of NR4A1 to the miR-224-5p promoter, the miR-224-5p promoter was predicted using the Promoter 2.0 prediction server, cloned, and inserted upstream of a luciferase open reading frame in the pGL3 promoter vector. A mutation within the binding sites between NR4A1 and miR-224-5p was generated using a QuikChange^®^ site-directed mutagenesis kit (Agilent, Palo Alto, CA, United States) according to the manufacturer’s recommendations. Luciferase activity was measured using the dual-luciferase assay system (Promega, Madison, WI, United States) with Top Count Microplate Scintillation Counter (Canberra, Meriden, CT, United States).

### Statistical Analysis

Data were expressed as the mean ± s.e.m. Statistical analysis was performed using SPSS 19.0 software. They were analyzed using one-way analysis of variance and least significant difference (LSD) *q* test using the SPSS 19.0 software package (IBM, Armonk, NY, United States). Statistical significance was defined as *P* < 0.05. ^∗^*P* < 0.05, and ^∗∗^*P* < 0.01.

## Results

### MiR-224-5p Was Increased in the Cerebral Cortex After MCAO and in Primary Cortical Neurons After OGD

**FIGURE 1:**
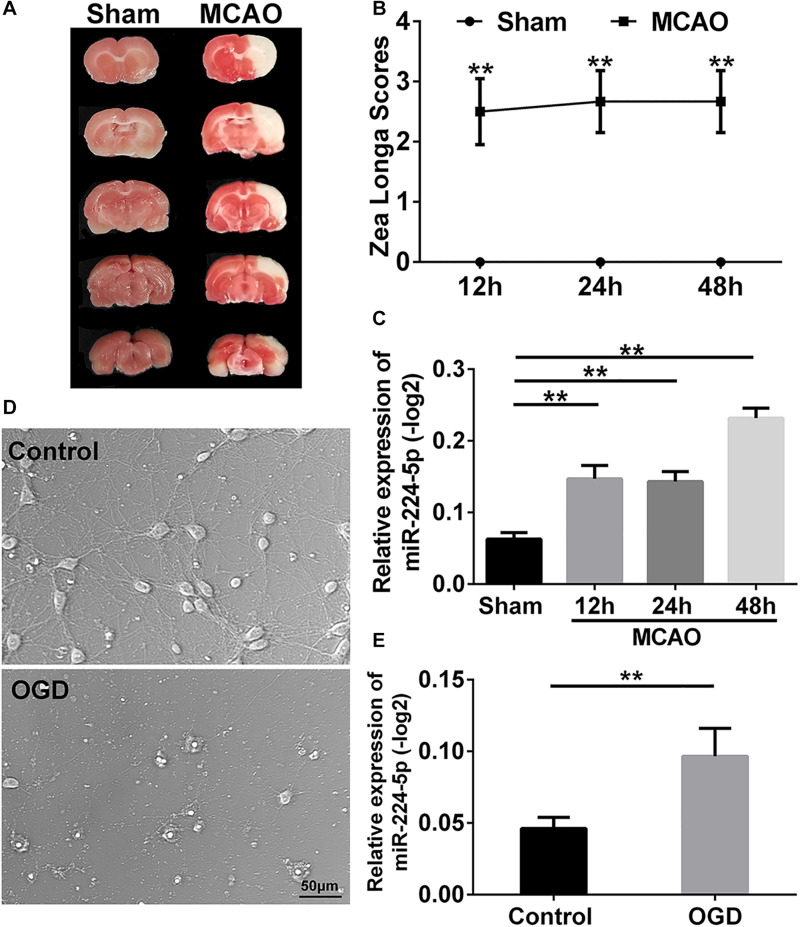
MiR-224-5p increased in the cerebral cortex after MCAO and neurons after OGD. **(A)** TTC staining showed the cerebral infarction after MCAO. The white area was indicated as cerebral infarction. **(B)** The Zea Longa scores were significantly increased in 12th, 24th, and 48th hours after MCAO compared with the sham group in rats (^∗^*^∗^P* < 0.01). **(C)** The expression of miR-224-5p increased in 12th, 24th, and 48th hours in rats after MCAO (^∗^*^∗^P* < 0.01). **(D)** The bright field images of neurons after OGD. **(E)** The expression of miR-224-5p was significantly increased in neurons after OGD (^∗∗^*P* < 0.01).

To investigate the mechanism of stroke, we established the MCAO model which was based on the suture method (Longa et al., 1989) in SD rats. 2 h after MCAO, the brain was harvested and stained with triphenyltetrazolium chloride (TTC) staining (Kramer et al., 2010). A white infarction area appeared in the MCAO group, while the brain tissue from rats in the sham group was red (Figure 1A). This indicated that the middle cerebral artery had been successfully blocked. Neurological deficit was evaluated using the Longa score (Longa et al., 1989). There was a statistically significant difference in the scores in 12th, 24th, and 48th hours after MCAO when compared with the sham group (*n* = 6, ^∗∗^*P* < 0.01, Figure 1B). These indicated that the MCAO model was successful established. Then, we obtain the tissue of ischemic penumbra of the brain. We found that miR-224-5p significantly increased in 12th, 24th, and 48th hours after MCAO in rats (*n* = 6, Figure 1C).

*In vitro*, we established the OGD model in primary cortical neurons. The bright field images were presented in Figure 1D. The number of cortical neurons was significantly decreased at 24 h after oxygen-glucose deprivation (OGD; Figure 1D). Then, we found that the expression of miR-224-5p was significantly increased at 24 h after OGD (*n* = 6, ^∗∗^*P* < 0.01, Figure 1E).

### Inhibition of MiR-224-5p Improved the Morphology and Increased the Number of Primary Cortical Neurons After OGD

**FIGURE 2:**
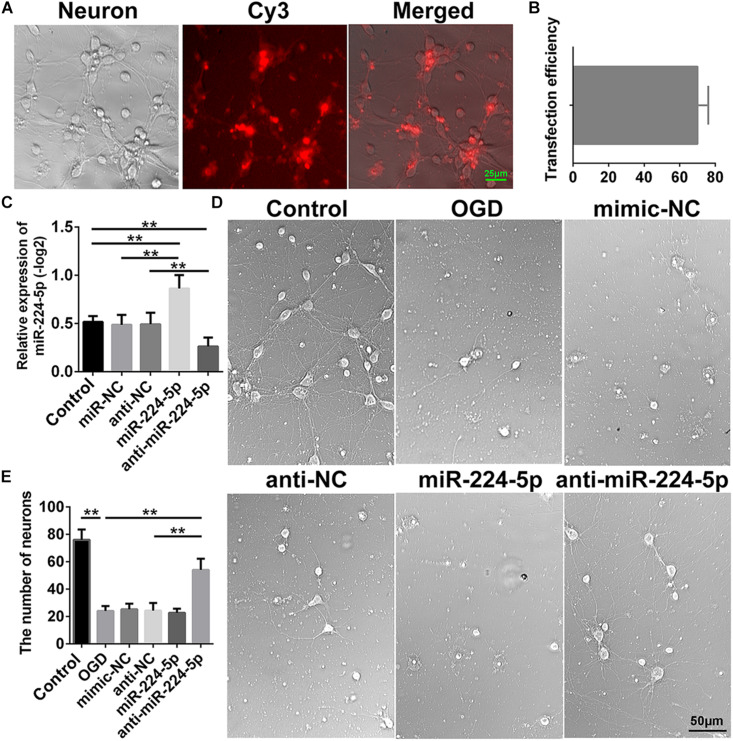
Inhibition of MiR-224-5p Improved the Morphology and Increased the Number of Primary Cortical Neurons After OGD. **(A)** The images of transfection of Cy3 in neurons. **(B)** The transfection efficiency of neurons. **(C)** The mimic of miR-224-5p could promote the expression of miR-224-5p, and the inhibitor of miR-224-5p could inhibit the expression of miR-224-5p (^∗∗^*P* < 0.01). **(D)** The images of primary cortical neurons in the control group, OGD group, mimic-NC group, anti-NC group, miR-224-5p group, and anti-miR-224-5p group. **(E)** Inhibition of miR-224-5p increased the number of neurons after OGD injury (^∗∗^*P* < 0.01).

In order to investigate the effect of miR-224-5p, we transfected the mimic and inhibitor of miR-224-5p into primary cortical neurons. Transfection of Cy3 is shown in Figure 2A. The transfection efficiency is shown in Figure 2B. Meanwhile, we found that the mimic of miR-224-5p could promote the expression of miR-224-5p and the inhibitor of miR-224-5p could inhibit the expression of miR-224-5p (*n* = 6, ^∗∗^*P* < 0.01, Figure 2C). Then, we showed the images of primary cortical neurons in the control group, OGD group, mimic-NC group, anti-NC group, miR-224-5p group, and anti-miR-224-5p group (Figure 2D). We found that the number of primary cortical neurons was decreased significantly after OGD compared with the control group (*n* = 6, ^∗∗^*P* < 0.01, Figure 2E). However, inhibition of miR-224-5p inhibited the number of apoptotic neurons after OGD injury.

### Inhibition of MiR-224-5p Ameliorated Neurodegeneration and Reduced the Apoptosis of Neurons

**FIGURE 3:**
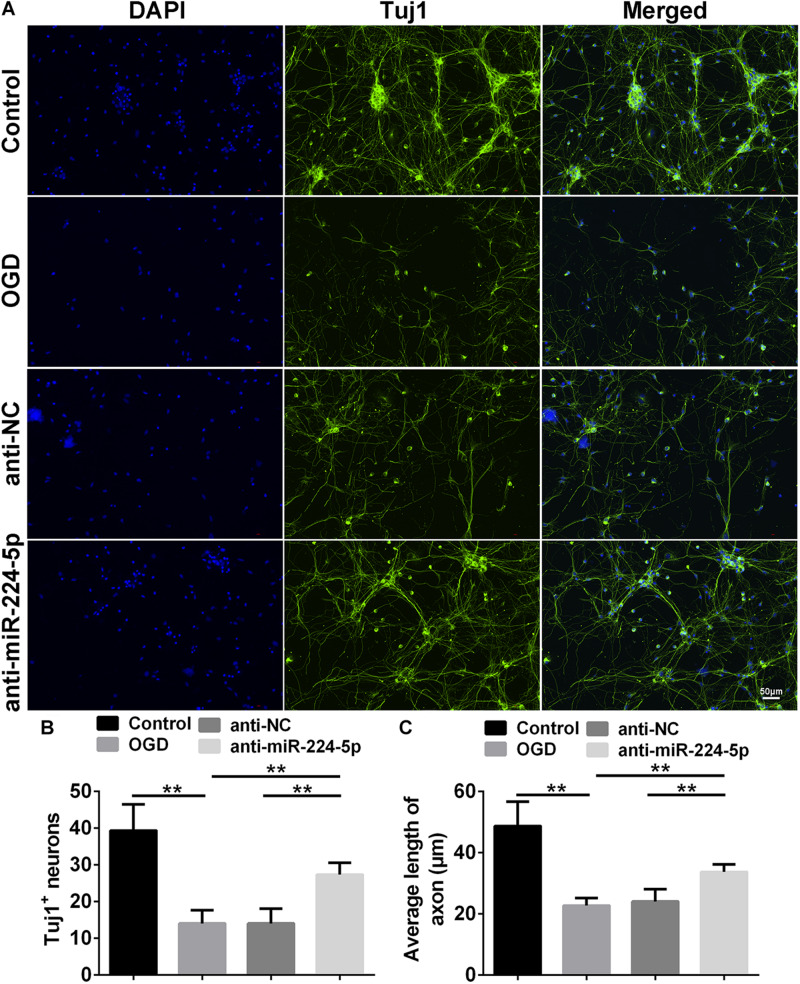
Inhibition of MiR-224-5p Ameliorated Neurodegeneration. **(A)** Tuj1^+^ neurons (green) were observed in control group, OGD group, anti-NC group, and anti-miR-224-5p group. Nuclei were visualized by DAPI (blue). **(B)** The number of Tuj1^+^ neurons was quantified. The number of Tuj1^+^ cells was increased in the anti-miR-224-5p group compared with anti-NC (*^∗∗^P* < 0.01). **(C)** The neurite length was also quantified. The axonal length of neurons was increased in the anti-miR-224-5p group compared with anti-NC (*^∗∗^P* < 0.01).

Tuj1^+^ cells were investigated in the control group, OGD group, anti-NC group, and anti-miR-224-5p group (Figure 3A). We found that the number of Tuj1^+^ cells was significantly reduced in the OGD group compared with the control group. However, the number of Tuj1^+^ cells was significantly increased in the anti-miR-224-5p group, when compared with the anti-NC group (*n* = 6, ^∗∗^*P* < 0.01, Figure 3B). Furthermore, the length of neuronal axons was significantly reduced in the OGD group compared with the control group. When compared with the anti-NC group, the average axonal length was significantly increased in the anti-miR-224-5p group (*n* = 6, ^∗∗^*P* < 0.01, Figure 3C). The above results showed that inhibition of miR-224-5p ameliorated neurodegeneration in OGD injury.

**FIGURE 4:**
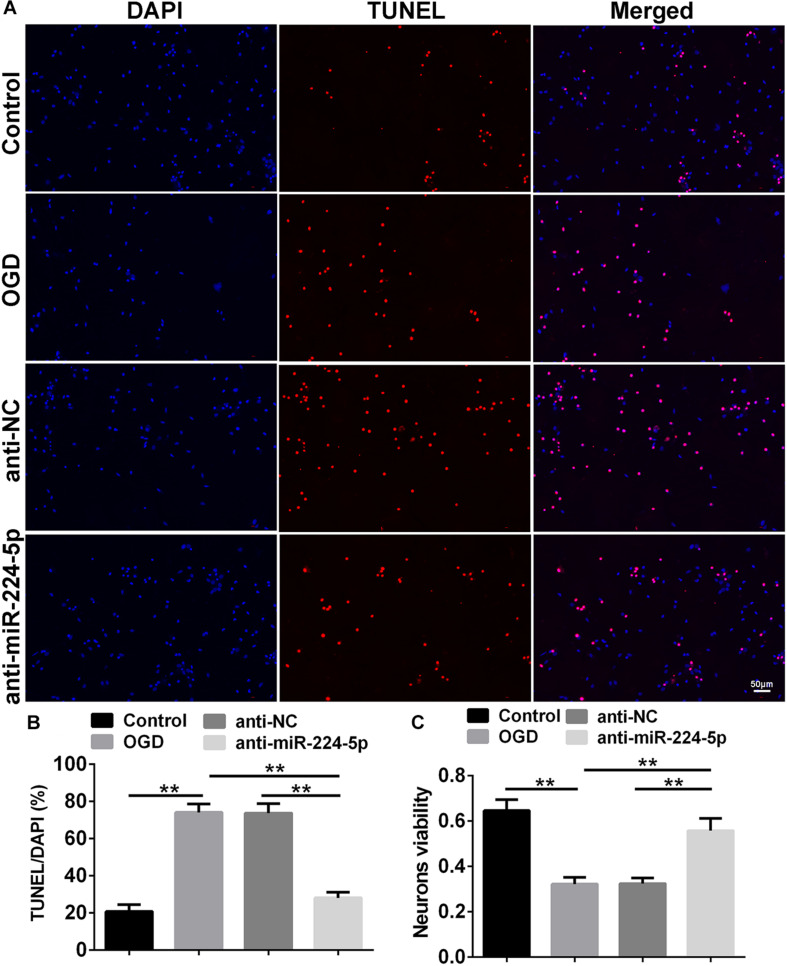
Inhibition of MiR-224-5p Reduced the Apoptosis of Neurons. **(A)** The apoptosis of neurons (red) were observed in the normal group, OGD group, anti-NC group, and anti-miR-224-5p group. Nuclei were visualized by DAPI (blue). **(B)** The apoptotic ratio of neurons was quantified. When compared with the anti-NC group, the apoptotic ratio of neurons significantly decreased in the anti-miR-224-5p group (^∗∗^*P* < 0.01). **(C)** The viability of neurons was quantified by MTT. When compared with anti-NC, the viability of neurons significantly increased in the anti-miR-224-5p group (^∗∗^*P* < 0.01).

TUNEL-positive cells were also observed in the control group, OGD group, anti-NC group, and anti-miR-224-5p group (Figure 4A). Apoptosis of primary cortical neurons was increased in the OGD group compared to the control group. When compared with the anti-NC group, the number of neuronal apoptosis was decreased in the anti-miR-224-5p group (*n* = 6, ^∗∗^*P* < 0.01, Figure 4B), which induced that inhibition of miR-224-5p could reduce the apoptosis of hypoxic neurons. Meanwhile, neuron viability was significantly increased in the anti-miR-224-5p group when compared with the anti-NC group (*n* = 6, ^∗∗^*P* < 0.01, Figure 4C).

### MiR-224-5p Regulated the Expression of NR4A1 in Hypoxic Neurons

**FIGURE 5:**
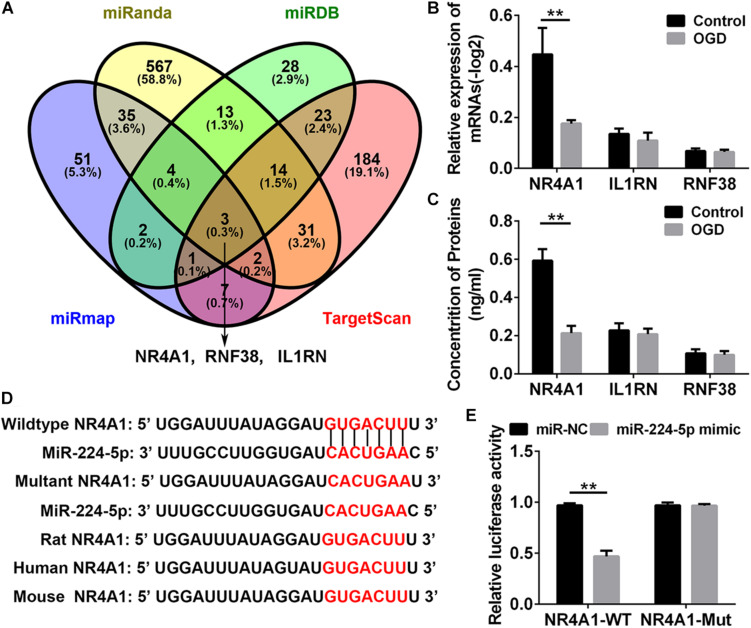
MiR-224-5p Regulated the Expression of NR4A1 in Hypoxic Neurons. **(A)** The intersection of the predicted target gene results of miR-224-5p in miRDB, TargetScan, miRanda, and miRmap. The different color represents the distinctive method of prediction. There were three target genes of miR-224-5p, including NR4A1, RNF38, and IL1RN. **(B)** QRT-PCR analyses revealed that the mRNA expression of NR4A1 was decreased in hypoxic neurons (*^∗∗^P* < 0.01). **(C)** ELISA analyses revealed that the protein expression of NR4A1 was decreased in hypoxic neurons (*^∗∗^P* < 0.01). **(D)** Alignment of the seed regions of miR-224-5p with 3′-UTR of NR4A1. The red color indicated the pairing bases. **(E)** The relative luciferase activity was significantly decreased after treatment with miR-224-5p, whereas the inhibitory effect of miR-224-5p was abolished in the plasmid containing the mutant 3′-UTRs of NR4A1.

To investigate the mechanism of miR-224-5p participating in the regulation of nerve injury, we searched for the target genes of miR-224-5p in primary cortical neurons. NR4A1, IL1RN, and RNF38 were identified target genes of miR-224-3p by four bioinformatics databases, such as miRDB^1^, TargetScan^2^, miRanda^3^, and miRmap^4^ and were selected for further investigation (Figure 5A). QRT-PCR and ELISA analyses revealed that the mRNA and protein expression of NR4A1, but not IL1RN and RNF38, was decreased in hypoxic neurons (*n* = 6, ^∗∗^*P* < 0.01, Figures 5B,C). The 3′-UTRs of NR4A1 were cloned into the pmi-R-RB-REPORT plasmid, and the predicted binding sites for miR-224-5p are as shown in Figure 5D. The mutated construct was generated using site-directed mutagenesis of a reporter containing the 3′-UTRs of the NR4A1 genes (Figure 5D). The relative luciferase activity was significantly decreased after treatment with miR-224-5p, whereas the inhibitory effect of miR-224-5p was abolished in the plasmid containing the mutant 3′-UTRs of NR4A1 (*n* = 6, ^∗∗^*P* < 0.01, Figure 5E).

### Inhibition of MiR-224-5p Reduced the Apoptosis of Neurons Through NR4A1

**FIGURE 6:**
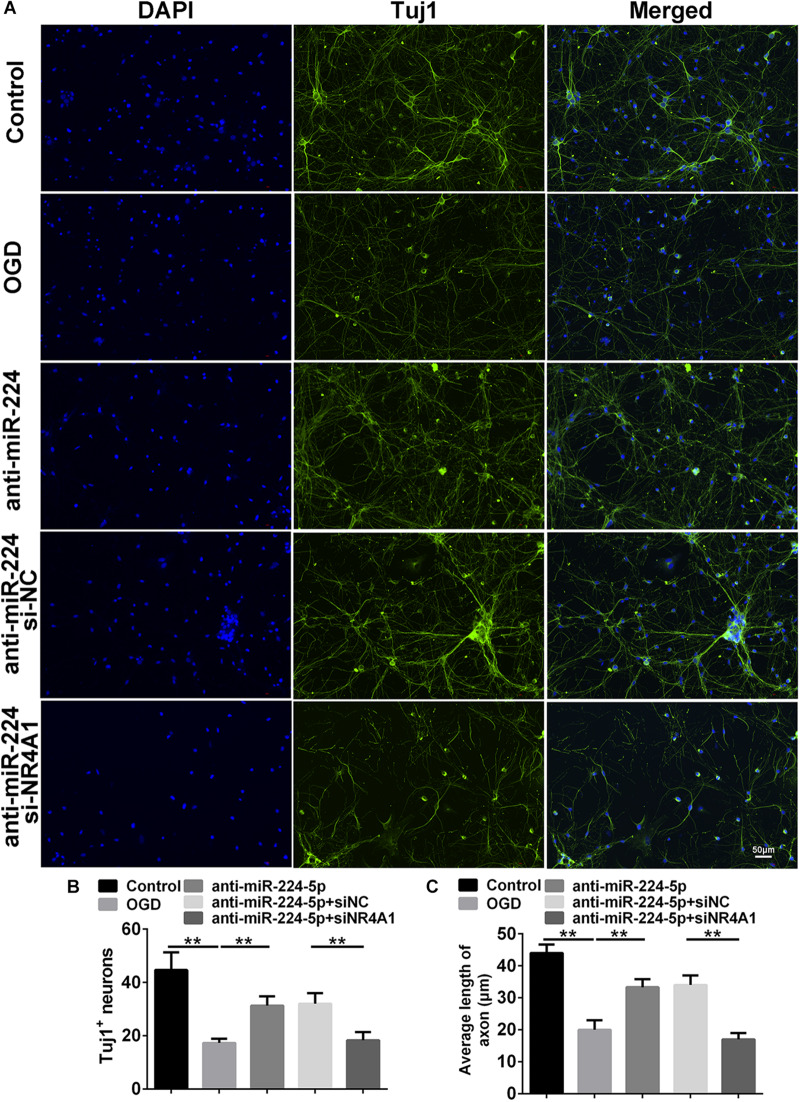
Inhibition of MiR-224-5p Ameliorated Neurodegeneration Through NR4A1. **(A)** Tuj1^+^ neurons (green) were observed in the control group, OGD group, anti-miR-224-5p group, anti-miR-224-5p + si-NC group, anti-miR-224-5p + si-NR4A1 group, and anti-NC + si-NR4A1 group. Nuclei were visualized by DAPI (blue). **(B)** The number of Tuj1^+^ neurons was quantified. The number of Tuj1^+^ cells was decreased in the anti-miR-224-5p + si-NR4A1 group compared with the anti-miR-224-5p + si-NC group (*^∗∗^P* < 0.01). **(C)** The neurite length was also quantified. The axonal length of neurons was decreased in the anti-miR-224-5p + si-NR4A1 group compared with the anti-miR-224-5p + si-NC group (*^∗∗^P* < 0.01).

**FIGURE 7:**
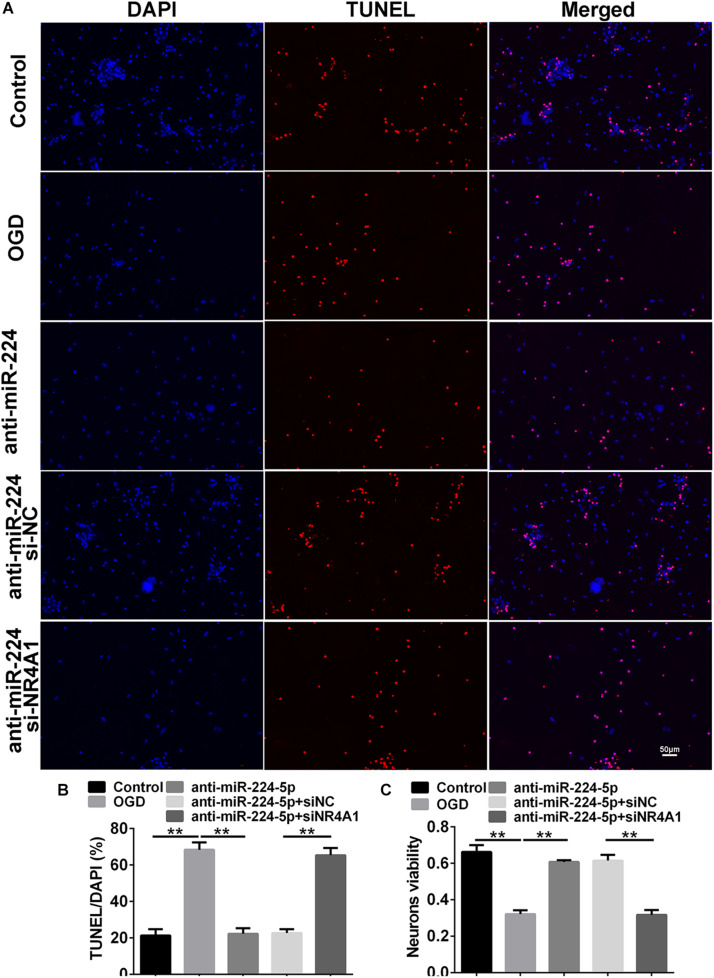
Inhibition of MiR-224-5p Reduced the Apoptosis of Neurons Through NR4A1. **(A)** The apoptosis of neurons (red) was observed in the control group, OGD group, anti-miR-224-5p group, anti-miR-224-5p + si-NC group, anti-miR-224-5p + si-NR4A1 group, and anti-NC + si-NR4A1 group. Nuclei were visualized by DAPI (blue). **(B)** The apoptotic ratio of neurons was quantified. The apoptotic ratio of neurons significantly increased in the anti-miR-224-5p + si-NR4A1 group compared with the anti-miR-224-5p + si-NC group (^∗∗^*P* < 0.01). **(C)** The viability of neurons was quantified by MTT. The viability of neurons was markedly decreased in the anti-miR-224-5p + si-NR4A1 group compared with the anti-miR-224-5p + si-NC group (^∗∗^*P* < 0.01).

We detected Tuj1^+^ cells and TUNEL^+^ cells in the control group, OGD group, anti-miR-224-5p group, anti-miR-224-5p + si-NC group, anti-miR-224-5p + si-NR4A1 group, and anti-NC + si-NR4A1 group (Figures 6A, 7A). The number of Tuj1 + cells was decreased in the anti-miR-224-5p + si-NR4A1 group compared with the anti-miR-224-5p + si-NC group (*n* = 6, ^∗∗^*P* < 0.01, Figure 6B). Meanwhile, after transfection of the miR-224-5p inhibitor and si-NR4A1, the average axonal length was significantly decreased compared with the anti-miR-224-5p + si-NC group (*n* = 6, ^∗∗^*P* < 0.01, Figure 6C). Apoptosis of primary cultured cortex neurons was increased in the anti-miR-224-5p + si-NR4A1 group compared to the anti-miR-224-5p + si-NC group (*n* = 6, ^∗∗^*P* < 0.01, Figure 7B). Meanwhile, the neuron viability was significantly decreased in the anti-miR-224-5p + si-NR4A1 group when compared with the anti-miR-224-5p + si-NC group (*n* = 6, ^∗∗^*P* < 0.01, Figure 7C). These results showed that NR4A1 was the potential target of miR-224-5p in ameliorating OGD injury.

## Discussion

In the present study, we found that the miR-224-5p inhibitor restrained neuronal apoptosis in OGD injury of neurons. Furthermore, we confirmed that nuclear receptor subfamily 4, group A, member 1 (NR4A1), was a direct target of miR-224-5p, which affected the neuroprotective effects of anti-miR-224-5p in hypoxic neurons in OGD.

As previously reported, stroke could lead to neurological dysfunction (Sun et al., 2010; Hu et al., 2017; Jain et al., 2017), and the process of stroke had the participation of neuronal apoptosis (Li et al., 1999). In this study, we found that brain infarction was obvious with TTC staining and Longa score confirmed the neurological deficit in MCAO rats. In recent years, accumulating evidence demonstrated that miRNAs played important roles in the pathogenesis of stroke. Meanwhile, hypoxia modulated miRNAs’ expression (Nallamshetty et al., 2013). Researchers found that miR-207 and miR-352 were downregulated after stroke. Moreover, miR-207 reduced the number of cellular lysosomes and autophagosomes and increased the number of autophagic vacuoles by downregulating lysosomal-associated membrane protein 2 (LAMP2), which attenuated cerebral ischemia mainly via affecting mitochondria-induced apoptosis (Tao et al., 2015). Inhibition of miR-134 improved OGD-induced ischemia-like injury in mouse N2A neuroblastoma cells by targeting heat shock protein A12B (HSPA12B; Chi et al., 2014). MiR-124 mediated stroke-induced neurogenesis, neuronal differentiation, and axonal outgrowth (Calderone et al., 2003; Cheng et al., 2009; Liu et al., 2011; Akerblom et al., 2012; Doeppner et al., 2013). These discoveries offered new avenues for the treatment of stroke. Therefore, the study on the mechanism of sparked neuronal apoptosis in cerebral hypoxia is the primary problem at present. In our study, we found that downregulation of miR-224-5p repressed OGD-induced neuronal hypoxic injury by preventing neuronal apoptosis. Previous studies had shown that miR-224-5p was induced under hypoxic conditions in melanoma and primary human trophoblasts (Mouillet et al., 2010; Hwang et al., 2014). Meanwhile, it had been reported that miR-224-5p enhanced cell proliferation and suppressed cell apoptosis in meningioma cells by targeting ERG2 (Wang et al., 2015). MiR-224-5p was also upregulated in esophageal squamous cell carcinoma; promoted cell proliferation, migration, and invasion; and suppressed cell apoptosis (He et al., 2015). Our finding demonstrated that the miR-224-5p inhibitor might suppress neuronal apoptosis, which was similar to the findings in the above studies. Therefore, the miR-224-5p suppressor might be regarded as a promoting factor for neural repair in stroke.

In this study, the miR-224-5p inhibitor prevented neuronal apoptosis. However, when we transfected si-NR4A1, the neuroprotective effect of the miR-224-5p inhibitor was blocked. NR4A1, a member of the NR4A family, performed its physiological effects through influencing protein expression, post-translational modification, and subcellular transposition (Fahrner et al., 1990; Hamers et al., 2013). It had a variety of biological functions and was involved in the regulation of cell proliferation, differentiation, development, apoptosis, immunity, and other biological processes (Fahrner et al., 1990). TNF could activate the transcription activity of the NR4A1 gene in macrophages and promote NR4A1 expression. Meanwhile, overexpression of NR4A1 in mouse macrophages could promote the expression of inflammatory factors, which promoted the apoptosis of cells (Hanna et al., 2012). In 2013, Xiao et al. (2013) found that NR4A1 ameliorated OGD-induced neural damage. In our study, we investigated the neuroprotection of NR4A1 *in vitro*. Then, we further confirmed that miR-224 was the upstream regulator of NR4A1. We found that the miR-224-5p inhibitor ameliorated OGD-induced neuronal apoptosis via targeting the 3′-UTR of NR4A1. This provides a new perspective in understanding the pathogenesis of neuronal OGD injury.

## Conclusion

In conclusion, we have found that the inhibitor of miR-224-5p inhibits apoptosis of neurons by regulating the expression of NR4A1 in the OGD of neurons. Therefore, miR-224-5p/NR4A1 can be an important regulatory mechanism for neuronal apoptosis in OGD injury of neurons.

## Data Availability Statement

All datasets generated for this study are included in the article/Supplementary Material.

## Ethics Statement

The animal study was reviewed and approved by Ethics Committee for Animal Experimentation of Shandong University.

## Author Contributions

X-WL conceived and designed the experiments. L-LL, SQ, M-LW, H-KW, Y-XS, and K-MW performed the experiments, analyzed the data, and wrote the manuscript. All authors contributed to the article and approved the submitted version.

## Conflict of Interest

The authors declare that the research was conducted in the absence of any commercial or financial relationships that could be construed as a potential conflict of interest.

## Footnotes

1 http://www.mirdb.org/

2 http://www.targetscan.org/vert_72/

3 http://www.microrna.org/microrna/microrna/home.do

4 http://mirmap.ezlab.org/app/

## Supplementary Material

FIGURE S1 The expression of NR4A1 was downregulated after transfection of siNR4A1 in neurons. **(A)** The result of qRT-PCR showed that NR4A1 was downregulated significantly after transfection of siNR4A1-1 and siNR4A1-2 in neurons (*^∗∗^P* < 0.01). **(B)** The result of WB showed that NR4A1 was downregulated significantly after transfection of siNR4A1-1 and siNR4A1-2 in neurons (^∗∗^*P* < 0.01).
