# Supplementary figures and images for: MiR224-5p Inhibitor Restrains Neuronal Apoptosis by Targeting NR4A1 in the Oxygen-Glucose Deprivation (OGD) Model
Source: Front Neurosci. 2020 Jun 25;14:613. doi: 10.3389/fnins.2020.00613 (PMC7330102; doi:10.3389/fnins.2020.00613)

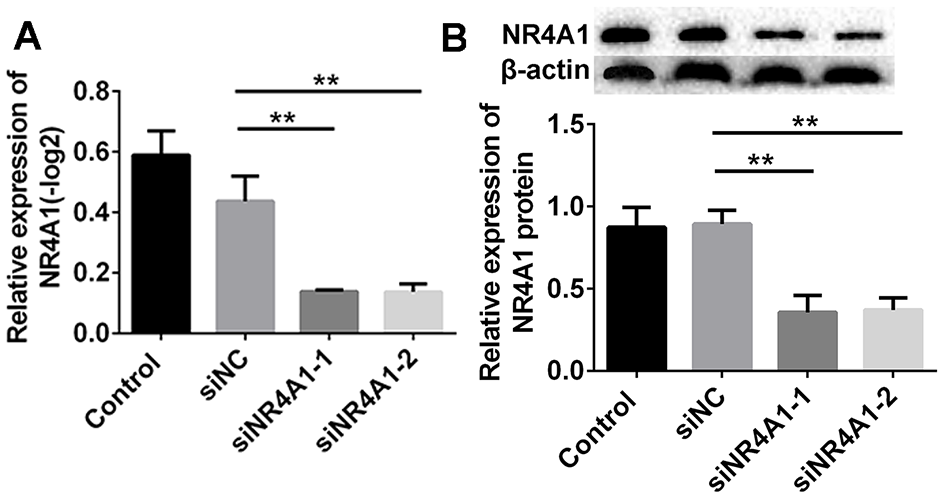

Supplement: FIGURE S1 — The expression of NR4A1 was downregulated after transfection of siNR4A1 in neurons. (A) The result of qRT-PCR showed that NR4A1 was downregulated significantly after transfection of siNR4A1-1 and siNR4A1-2 in neurons (∗∗P < 0.01). (B) The result of WB showed that NR4A1 was downregulated significantly after transfection of siNR4A1-1 and siNR4A1-2 in neurons (∗∗P < 0.01). [file Image_1.TIF]
